# Metabolomic analysis of dynamic response and drug resistance of gastric cancer cells to 5-fluorouracil

**DOI:** 10.3892/or.2012.2182

**Published:** 2012-12-11

**Authors:** SHINSUKE SASADA, YOSHIHIRO MIYATA, YASUHIRO TSUTANI, NAOHIRO TSUYAMA, TSUTOMU MASUJIMA, JUN HIHARA, MORIHITO OKADA

**Affiliations:** 1 Department of Surgical Oncology, Research Institute for Radiation Biology and Medicine, Hiroshima University, Hiroshima, Japan; 2 Analytical Molecular Medicine and Devices Laboratory, Graduate School of Biomedical and Health Sciences, Hiroshima University, Hiroshima, Japan

**Keywords:** metabolomics, mass spectrometry, gastric cancer, fluorouracil, amino acid, proline dehydrogenase

## Abstract

Metabolomics has developed as an important new tool in cancer research. It is expected to lead to the discovery of biomarker candidates for cancer diagnosis and treatment. The current study aimed to perform a comprehensive metabolomic analysis of the intracellular dynamic responses of human gastric cancer cells to 5-fluorouracil (5-FU), referencing the mechanisms of drug action and drug resistance. Small metabolites in gastric cancer cells and 5-FU-resistant cells were measured by liquid chromatography-mass spectrometry. Candidates for drug targets were selected according to the presence or absence of resistance, before and after 5-FU treatment. In addition, the gene expression of each candidate was assessed by reverse transcription-polymerase chain reaction. The number of metabolites in cancer cells dramatically changed during short-term treatment with 5-FU. Particularly, proline was reduced to one-third of its original level and glutamate was increased by a factor of 3 after 3 h of treatment. The metabolic production of glutamate from proline proceeds by proline dehydrogenase (PRODH), producing superoxide. After 5-FU treatment, PRODH mRNA expression was upregulated 2-fold and production of superoxide was increased by a factor of 3. In 5-FU-resistant cells, proline and glutamate levels were less affected than in non-resistant cells, and PRODH mRNA expression and superoxide generation were not increased following treatment. In conclusion, the authors identified a candidate biomarker, PRODH, for drug effects using a metabolomic approach, a result that was confirmed by conventional methods. In the future, metabolomics will play an important role in the field of cancer research.

## Introduction

Gastric cancer is common in Asia, South America and Eastern Europe, accounting for more than 800,000 new cases per year worldwide. It is also the second most common cause of cancer-related deaths globally (1). Several chemotherapy regimens have been established as the first-line therapy in the treatment of advanced gastric cancer (2–4). Nevertheless, the effectiveness of gastric cancer chemotherapy is limited, compared with similar treatment for other malignancies, such as colorectal cancer and breast cancer. Therefore, in order to improve the effectiveness of chemotherapy for this cancer, it is important to investigate the cellular responses and resistance mechanisms associated with anticancer agents.

To investigate these topics, we turned to metabolomics, which is considered the third pillar of systems biology, after genomics and proteomics, representing the end of the biochemical cascade (5). Well-recognized tools of metabolomics include gas chromatography-mass spectrometry (6), liquid chromatography-mass spectrometry (LC-MS) (7) and nuclear magnetic resonance (8). This is a novel perspective, and the comprehensive investigation of metabolic alterations in malignancies has rarely been conducted, in contrast to the well-studied genomics and proteomics fields. Moreover, only a few experiments investigating the cellular responses to chemotherapy have been reported (9–11).

Herein, we applied the LC-MS method to investigate the cellular response of gastric cancer cells to 5-fluorouracil (5-FU). We observed short-term reactions and the dynamic responses to assess the effect of the anticancer agent. In addition, we established a 5-FU-resistant cell line, MKN45/F2R, by subjecting the human gastric cancer cell line MKN45 to continuous 5-FU exposure (12). We also investigated the mechanisms of drug resistance, comparing 2 cell lines, and identified a factor, proline dehydrogenase (PRODH), that induces superoxide and is involved in the action and resistance of 5-FU. This is the first study highlighting a short-term, cellular, dynamic response of gastric cancer cells and the resistance mechanism to an anticancer agent by using the metabolomics approach.

## Materials and methods

### Chemicals and solutions

High-performance liquid chromatography (HPLC)-grade methanol and formic acid were purchased from Kanto Chemical (Tokyo, Japan). 5-FU was purchased from Kyowa Hakko (Tokyo, Japan).

### Cell culture and treatment

Human poorly differentiated gastric adenocarcinoma MKN45 cells were purchased from the American Type Culture Collection and maintained in RPMI-1640 medium, supplemented with 10% fetal bovine serum, 100 U/ml penicillin, and 100 μg/ml streptomycin in a humidified atmosphere of 5% CO_2_ at 37°C. 5-FU-resistant MKN45/F2R cells were maintained in a culture medium containing 2 μM 5-FU. To eliminate the effects of 5-FU in our experiments, the resistant cells were cultured in a drug-free medium for at least 2 weeks before any procedure. Each cell line was planted in 6-well plates at a density of 1×10^6^ per well. Cells were exposed to 10 μM 5-FU or medium alone (control group, 0 h). At specified times (up to 12 h after the onset of treatment), cells were rinsed 3 times with phosphate-buffered saline (PBS) and harvested by trypsinization.

### Cell proliferation assay

Gastric cancer cells were seeded onto 96-well plates (1×10^4^ cells/well). After 24 h, the cells were treated with various concentrations of 5-FU in 100 μl medium and incubated at 37°C in an atmosphere of 5% CO_2_. After the 72-h incubation period, CellTitler 96^®^ Aqueous One Solution (Promega, Madison, WI, USA) was added (20 μl/well) and the resultant mixture was incubated for 1 h at 37°C in an atmosphere of 5% CO_2_. Then, the absorbance at 490 nm with a reference at 650 nm was measured with an Emax microplate reader (Molecular Devices, Tokyo, Japan).

### LC-MS

Harvested cells were transferred to a 1.5-ml conical tube and spun down. Cells were then washed with PBS once. Excess PBS was removed by aspiration, and the cell pellets were suspended in methanol (100 μl/10^6^ cells), followed by sonication. The samples were then centrifuged at 10,000 × g at 4°C for 10 min, and supernatants were dispensed and stocked at −40°C.

For LC separation, a binary gradient HPLC system consisting of an LC-20AD pump coupled to an SIL-20AC autosampler (Shimadzu, Kyoto, Japan) with the sample cooler set at 4°C was used. Chromatographic reversed-phase separations were performed on an L-column2 ODS (1.5×150 mm, 5 μm) (CERI, Tokyo, Japan), operated at room temperature. The mobile phases used consisted of purified water containing 0.1% formic acid as solvent A, and methanol containing 0.1% formic acid as solvent B. The flow-rate gradient and buffer composition were produced as follows: 0–1 min, held at 3% solvent B using a flow rate of 60 μl/min; 1–3 min, linear flow rate gradient from 60 to 20 μl/min; 3–6 min, held at 20 μl/min; 6–16 min, linear gradient of buffer composition from 3 to 99% solvent B and of flow rate from 20 to 60 μl/min; 16–45 min, held at 99% solvent B; 45–57 min, back to 3% solvent B and re-equilibrated for 12 min using a flow rate of 170 μl/min.

Mass spectrometry experiments were carried out on a QSTAR Elite, a hybrid quadrupole time-of-flight instrument attached to a Turboionspray electrospray ionization source (AB SCIEX, Framingham, MA, USA) working on positive-ion mode. The LC eluate was ionized at a spray voltage of 4.5 kV. We used information-dependent acquisition in data-acquisition software Analyst QS to obtain both full-scan spectra of *m/z* 50–1000 and MS/MS spectra of automatically detected peaks. Then, mass spectra were analyzed using Marker View software version 1.2 (AB SCIEX) to perform peak extraction, data alignment with LC retention time, normalization by total peak area, and statistical analyses.

### Reverse transcription-polymerase chain reaction (RT-PCR)

Total RNA was isolated using an RNeasy Mini kit (Qiagen, Chatsworth, CA, USA) and reverse transcribed with a Transcriptor First-Strand cDNA Synthesis kit (Roche, Tokyo, Japan). PCRs were performed with KOD FX (Toyobo, Osaka, Japan) and GeneAmp PCR System 9700 (Applied Biosystems, Tokyo, Japan). The specific primers for PRODH were: forward, 5′-CGGAGAGCAGGAGCAGAGGCTTTGA-3′; reverse, 5′-GCCGTGGACAGCGGGACGAA-3′. Glyceraldehyde-3-phosphate dehydrogenase (GAPDH) was amplified as a loading control with the following primers: forward, 5′-ACCA CAGTCCATGCCATCAC-3′; reverse, 5′-TCCACCACCC TGTTGCTGTA-3′. The PCR profile was 94°C for 2 min, 35 cycles at 98°C for 10 sec, 60°C for 30 sec, and 68°C for 30 sec. All reaction products (10 μl with glycerol loading buffer) were run on a 1% agarose gel and stained with ethidium bromide. Data were normalized to GAPDH.

### Reactive oxygen species (ROS)/superoxide measurement

To measure intracellular ROS and superoxide, a Total ROS/Superoxide detection kit (Enzo Life Sciences, Farmingdale, NY, USA) was used. Cells were stained for 30 min at 37°C in the dark with 1 μM of ROS and superoxide-sensitive fluorescent dyes, and subsequently assayed by flow cytometry FACSAria II (BD Biosciences, San Jose, CA, USA). Double staining with fluorescein isothiocyanate and phycoerythrin was carried out, and data were assessed by FlowJo software version 7.6 (Tomy Digital Biology Co., Tokyo, Japan).

### Statistical analyses

Data for metabolites and mRNA expression were calculated and expressed as the mean ± standard deviation (SD) from at least 3 experiments. Statistical analysis of LC-MS data on Marker View 1.2 consisted of principal component analysis (PCA) with Pareto scaling and Student's t-test. For the mRNA experiments, a t-test was performed using Microsoft Excel.

## Results

### Drug sensitivity of gastric cancer cell lines

**Figure 1:**
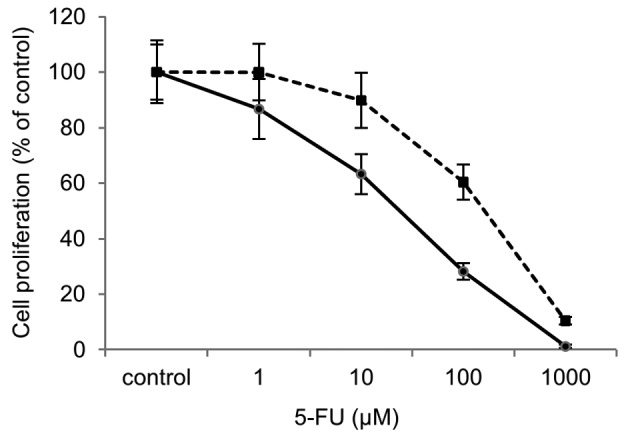
*In vivo* sensitivity of MKN45 (●) and MKN45/F2R (■) cells to 5-FU. Cells were cultured with various concentrations of 5-FU for 72 h. The relative number of living cells was measured, with each data point representing the mean ± SD.

MKN45/F2R cells were more resistant to 5-FU than were MKN45 cells (Fig. 1). The IC_50_ of MKN45 and MKN45/F2R cells were 23.8 and 161 μM, respectively. Growth in the absence of 5-FU was not appreciably different between the 2 cell lines (data not shown). In subsequent experiments, a drug concentration of 10 μM was used to examine the effects of a larger difference.

### Metabolome analysis

**Figure 2:**
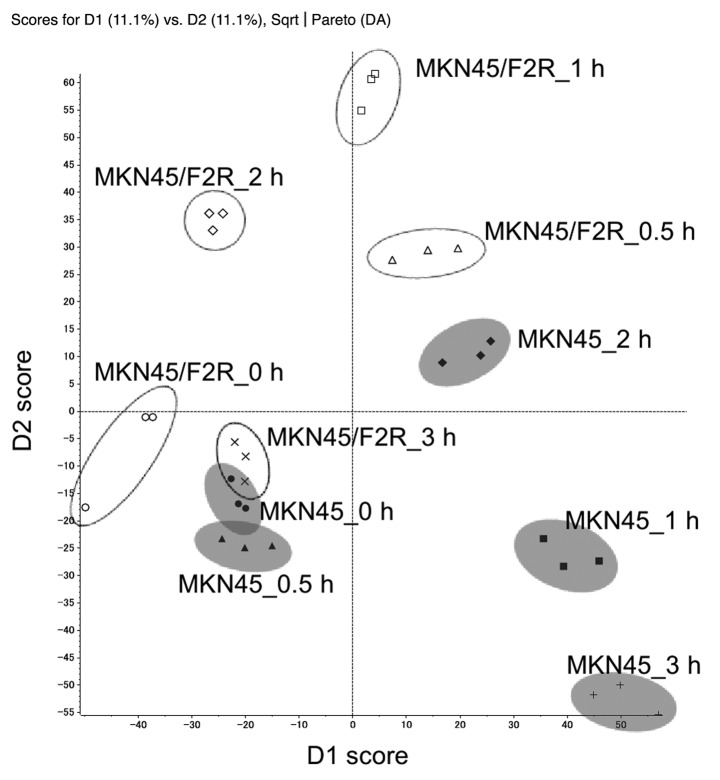
Scores plot from PCA of LC-MS data for MKN45 and MKN45/F2R cells. Each time-period group was separated from the other groups. Gray circles, MKN45 cells; void circles, MKN45/F2R cells.

The LC-MS analysis yielded the raw data, detecting 7107 (MKN45) and 5696 (MKN45/F2R) peaks. Among these, the numbers of peaks that increased by more than double their original size after 5-FU treatment were 642 (MKN45) and 140 (MKN45/F2R), and the numbers of peaks that decreased to less than half of their original size were 285 (MKN45) and 18 (MKN45/F2R). The identified metabolites are listed in Table I. Most of the peaks were identified as endogenous metabolites, such as amino acids, organic acids, lipids, and purines. To determine whether these spectra contained unique peaks that could differentiate the metabolic responses of the 2 cell lines, PCA was performed (Fig. 2). Each treatment-period group was clustered and separated from other groups, and the resultant data indicated the characteristic metabolic profiles of the cellular responses to 5-FU.

**Figure 3:**
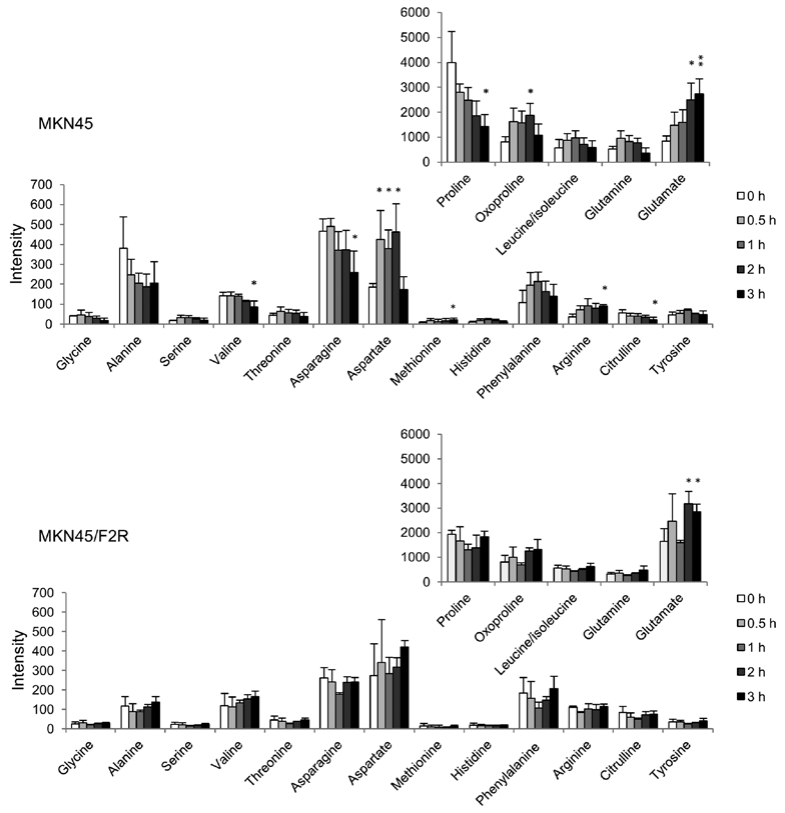
Metabolite profiling of MKN45 and MKN45/F2R cells. Time course validation of major amino acids was measured by LC-MS analysis, with each data point representing the mean ± SD. ^*^P<0.05; ^**^P<0.01 are for comparison of 5-FU-treated cells vs. untreated cells.

We then attempted to identify the responsible molecules that could serve as markers for 5-FU response. For those differential molecules, a time course validation of major amino acids is shown in Fig. 3. We found that the fluctuation for MKN45/F2R was less than that for MKN45 cells. In MKN45, the amino acids increasing at 3 h were glutamate, methionine and arginine, while those decreasing at 3 h were proline, valine, asparagine and citrulline. The transiently altered amino acids were oxoproline and aspartate. On the other hand, in MKN45/F2R cells, only glutamate increased at 3 h. In particular, proline and glutamate were very abundant and closely related in this metabolic pathway. In MKN45 cells, proline was reduced to one-third of its original value, and glutamate was increased to 3 times its original value after 3 h of treatment. These results suggest that the metabolic enzymes associated with the differential metabolites played an important role in the response to treatment and may be behind the mechanism of resistance to 5-FU. Among these enzymes, PRODH is particularly important; it catalyzes the first and the rate-limiting step of 2 reactions converting proline to glutamate at the mitochondria, and the catabolism of proline produces superoxide.

### Upregulation of PRODH mRNA and ROS/superoxide generation after 5-FU treatment

**Figure 4:**
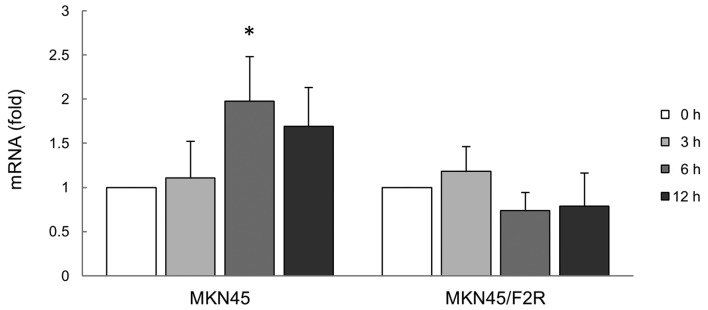
The fluctuation of PRODH mRNA expression levels in response to 5-FU stimulation was measured by RT-PCR. The graphs show the densitometric analysis of the relative expression of PRODH mRNA normalized to GAPDH. ^*^P<0.05 is for comparison of 5-FU-treated cells vs. untreated cells.

We then investigated the amount that PRODH gene expression was altered in response to 5-FU stimulation. A significant increase in the concentration of PRODH mRNA was observed in MKN45 cells after 6 h of treatment (Fig. 4). The expression of PRODH mRNA peaked after 6 h of treatment, resulting in a 2-fold increase in the PRODH transcript level. In MKN45/F2R cells, PRODH mRNA expression did not vary over time.

**Figure 5:**
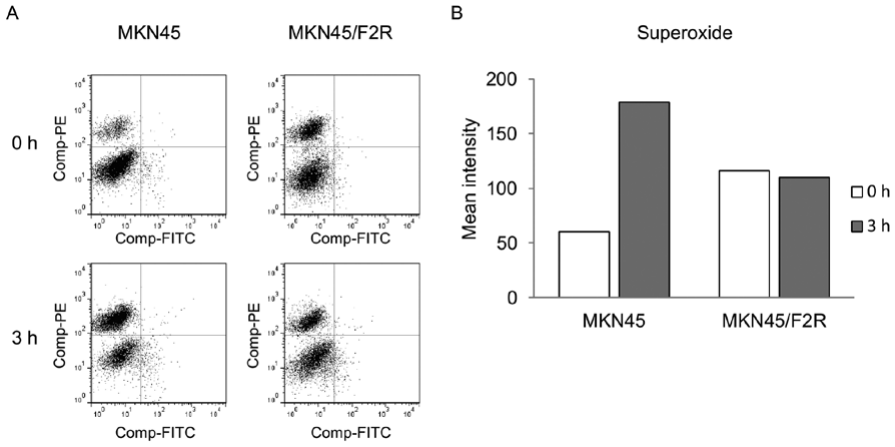
ROS/superoxide assay by flow cytometry. (A) ROS was measured with fluorescein isothiocyanate (FITC) and superoxide with phycoerythrin (PE). Significant numbers of superoxide-producing cells were observed in the MKN45 cells following 5-FU treatment. (B) The mean fluorescence intensity of superoxide in MKN45 and MKN45/F2R cells is shown in the histogram.

In general, proline catabolism first produces a superoxide anion with the chemical formula O_2_^−^, which is subsequently changed into a variety of ROS. Therefore, we not only highlighted ROS, but also superoxide. We found that both MKN45 and MKN45/F2R cells produced more superoxide than ROS, and MKN45/F2R produced more superoxide than MKN45 at the baseline. In MKN45 cells, the shift to the upper left quadrant was recognized, indicating the increased superoxide generation. In MKN45 cells, the mean fluorescence intensity of superoxide increased 3-fold following 3 h of treatment, while in MKN45/F2R, the intensity did not increase (Fig. 5).

## Discussion

In this study, we focused on small-molecule metabolites to investigate the characteristics of cancer cells and the cellular response to 5-FU. While there have been some metabolomic reports on the comparison between cancer and normal tissue (13), cancer metastasis (14), and the cancer response to chemotherapy (9–11), to our knowledge, this is the first metabolomics-based study of the short-term, cellular, dynamic response of gastric cancer cells and the resistance mechanisms to an anticancer agent.

A previous study proposed a metabolic workflow for the LC-MS/MS platform (15). In this scheme, the first step is sample handling, followed by analytical treatment, quality assessment and multivariate analysis, data reduction by principal component variable grouping and groups interpretation, listing of candidates (biomarkers), and finally, biomarker identification. We followed virtually the same workflow with our method. Candidates were searched in the metabolomics database KEGG (16), and MS/MS spectral searches were carried out on MassBank (17). Since even mass accuracy below 1 ppm would generate large lists of potential biomarkers, further MS/MS data were mandatory for structural confirmation. In general, multivariate analysis plays a key role in metabolomics for the comparison of large sets of data from various samples. In the case of LC-MS, metabolomics data processing generates a large amount of features consisting of retention time and *m/z*. Pattern recognition tools, such as PCA, show covariation between metabolites, with metabolism global trends retaining the largest part of the information (18).

Previously, researchers have focused more on cancer metabolism because of the realization that cancer genomics can be understood on the basis of metabolic pathways (19,20). However, little attention has been given to amino acids as substrate for energetics or as mediators of cellular signaling (21). Tumor cells require large amounts of nutrients, including amino acids, for rapid growth and uncontrolled proliferation, leading to perturbations in the amino acid metabolism of cancer (22).

In this study, the intracellular metabolites of gastric cancer cells dynamically changed within a few hours following 5-FU treatment. In particular, several amino acids were dramatically up or downregulated. Among these, we highlighted 2, proline and glutamate, since they were abundant and were continuously altered in MKN45 cells following 5-FU treatment. Additionally, the percentage of amino acids in the medium remained virtually unchanged after treatment (data not shown). This indicates that both proline and glutamate were metabolized within cells instead of being taken up into cells.

We additionally analyzed the molecule PRODH, also known as proline oxidase, a mitochondrial inner membrane enzyme that catalyzes the first step of proline degradation (23). In this process, proline is converted to glutamate through an intermediate, pyrroline-5-carboxylate (P5C). The function of PRODH is to catalyze the rate-limiting, 2-electron oxidation of proline to P5C, and the pair of electrons can then be used for direct reduction of oxygen to form superoxide (24). The superoxide is then transformed to ROS, a molecule related to cell death. PRODH has been found to be upregulated by p53, peroxisomal proliferator-activated receptor γ, and metabolic stress (21,25,26). Although PRODH expression levels were found to be much lower in several types of cancers, including stomach cancer, as compared to normal tissues (27), the relationship between PRODH and the anticancer effect of 5-FU has yet to be elucidated.

The present study demonstrated the upregulation of PRODH mRNA expression and superoxide generation in the gastric cancer cell line MKN45 following 5-FU treatment. This result indicates that 5-FU induces the PRODH gene directly or indirectly and stimulates the generation of mitochondrial superoxide, following cell death. In fact, PRODH mRNA expression was slower than the production of superoxide. In one potential explanation for these results, PRODH activity might first be stimulated by 5-FU, and the secondary gene expression might then be induced. The PRODH enzyme activity may be expressed as the ratio of proline to P5C. However, in this study, P5C could not be fully detected. The explanation for this result was thought to be that P5C was present at too small levels to be detectable or that it was an acidic substance. Thus, more sensitive measurements or the negative ion mode assay may be necessary.

Another mechanism of cell death may involve hypoxia-inducible factor-1α (HIF-1α) and vascular endothelial growth factor (VEGF) (21,27). PRODH produces P5C, which can then be converted to glutamate and α-ketoglutarate (α-KG). Since α-KG is not only a central substrate of the tricarboxylic acid cycle, but also a critical substrate for prolyl hydroxylase (PHD), HIF-1α may be downregulated by PHD activity. The expression levels of HIF-1α and its downstream gene, VEGF, have been shown to be reduced by PRODH (27).

We also investigated the mechanism of resistance to 5-FU using 5-FU-resistant cells. Specifically, we studied MKN45/F2R, which is a 5-FU-resistant cell line derived from MKN45 gastric cancer cells (12). The decrease in orotate phosphoribosyltransferase (OPRT), which metabolizes 5-FU to 5-fluorouridine monophosphate, plays an important role in the resistance to 5-FU chemotherapy (12). However, MKN45/F2R cells show cross-resistance to other agents, such as taxanes, platinum agents and SN38. In addition, OPRT-knockout MKN45 cells were found to be more resistant than were MKN45 cells (12). This indicates that other factors correlate with drug resistance. Although several candidate genes were identified, the mechanism of resistance is not well known. In the current metabolome analysis, fewer amino acids in MKN45/F2R cells showed change than in MKN45 cells following 5-FU treatment. This lower rate of effect can be traced to drug resistance. Since PRODH mRNA expression and superoxide generation in MKN45/F2R cells were not upregulated following 5-FU treatment, we can conclude that PRODH may be involved in drug resistance. The present study suggests that the inhibition of DNA or RNA synthesis by 5-FU and the resultant genetic stress induces PRODH activity following mitochondrial superoxide generation, represented as changes in metabolites.

The metabolomics approach to cancer research is still in a challenging phase despite advances in metabolomic techniques used in various scientific fields. We performed a metabolomic analysis of the effect of 5-FU treatment on human gastric cancer cells, including a detailed study of the mechanism of action of the agent. In the future, confirmation of the prominent metabolic pathways deduced from metabolomics should be achieved by enzyme tests, reverse transcriptomics, or genomics (11). Our results were consistent with metabolomic and reverse transcriptomic analyses, and show that metabolomics can serve as an important tool in cancer research.

## Figures and Tables

**Table I:** Metabolites identified from gastric cancer cells by LC-MS/MS study.

Molecule	Formula	*m/z*	RT (min)	MS fragments *(m/z)*
Glycine	C_2_H_5_NO_2_	76.04	6.09	
Alanine	C_3_H_7_NO_2_	90.05	6.23	
Serine	C_3_H_7_NO_3_	106.05	6.16	60
Creatinine	C_4_H_7_N_3_O	114.07	5.7	
Proline	C_5_H_9_NO_2_	116.07	6.64	70
Guanidinoacetate	C_3_H_7_N_3_O_2_	118.06	6.06	72, 76, 101
Valine	C_5_H_11_NO_2_	118.09	6.6	72
Threonine	C_4_H_9_NO_3_	120.07	6.31	56, 74, 102
Cysteine	C_3_H_7_NO_2_S	122.03	6.43	76
Oxoproline	C_5_H_7_NO_3_	130.05	6.42	84
Creatine	C_4_H_9_N_3_O_2_	132.08	6.36	90
Leucine/Isoleucine	C_6_H_13_NO_2_	132.10	11.22	86
Asparagine	C_4_H_8_N_2_O_3_	133.06	6.45	74, 87, 116
Aspartate	C_4_H_7_NO_4_	134.04	6.57	70, 74, 88
Adenine	C_5_H_5_N_5_	136.06	6.6	81, 119
4-Guanidinobutanoate	C_5_H_11_N_3_O_2_	146.09	6.48	
γ-Butyrobetaine	C_7_H_15_NO_2_	146.11	5.7	60, 87
Glutamine	C_5_H_10_N_2_O_3_	147.08	6.25	84, 130
Glutamate	C_5_H_9_NO_4_	148.06	6.4	84, 102, 130
Methionine	C_5_H_11_NO_2_S	150.06	8.76	104, 133
Histidine	C_6_H_9_N_3_O_2_	156.08	5.67	110
Carnitine	C_7_H_15_NO_3_	162.11	5.7	60, 85, 103
Phenylalanine	C_9_H_11_NO_2_	166.09	18.47	120
Arginine	C_6_H_14_N_4_O_2_	175.12	5.71	60, 70, 116, 130, 158
Citrulline	C_6_H_13_N_3_O_3_	176.10	6.26	70, 113, 159
Tyrosine	C_9_H_11_NO_3_	182.08	11.53	136, 165
Phosphocholine	C_5_H_15_NO_4_P	184.07	6.24	86, 125
Glucose	C_6_H_12_O_6_	203.05	6.08	
Acetylcarnitine	C_9_H_18_NO_4_	204.12	6.62	85, 145
Propionylcarnitine	C_10_H_19_NO_4_	218.13	6.74	85, 159
Pantothenate	C_9_H_17_NO_5_	220.12	26.68	72, 90, 202
Lumichrome	C_12_H_10_N_4_O_2_	243.09	31.87	172, 198
L-Argininosuccinate	C_10_H_18_N_4_O_6_	291.13	6.28	
5′-Methylthioadenosine	C_11_H_15_N_5_O_3_S	298.10	28.11	136
Ketosphingosine	C_18_H_35_NO_2_	298.27	33.07	282
Sphingosine	C_18_H_37_NO_2_	300.29	33.67	252, 282
Sphinganine	C_18_H_39_NO_2_	302.31	33.97	
Glutathione	C_10_H_17_N_3_O_6_S	308.09	9.98	179
N-Acetylneuraminate	C_11_H_19_NO_9_	310.11	8.76	121, 167, 274, 292
Phytosphingosine	C_18_H_39_NO_3_	318.30	33.6	282
Palmitoylcarnitine	C_23_H_45_NO_4_	400.34	35.55	85

*m/z*, mass to charge ratio; RT, retention time; MS, mass spectrometry.
